# Supplementary material for: Digital security vulnerabilities and threats implications for financial institutions deploying digital technology platforms and application: FMEA and FTOPSIS analysis
Source: PeerJ Comput Sci. 2021 Aug 3;7:e658. doi: 10.7717/peerj-cs.658 (PMC8356653; doi:10.7717/peerj-cs.658)
Supplement: Supplemental Information 2 [file peerj-cs-07-658-s002.pdf]

QUESTIONNAIRE

Dear Participant,

INFORM CONSENT

This research seeks to understand the antecedents of Digital Innovation deployment on digital security risk management: an evaluation of digital security risk with Failure Mode Effect Analysis (FMEA) and Fuzzy AHP techniques. Digital security risks aspects are a combination of vulnerabilities from the digital platforms, physical environment, the people and the organisation within the digital ecosystems. The outcomes of this research are to exude the various level of digital security vulnerabilities by deploying and usage of Big Data Analytics (BDA), Internet of Things (IoTs) and Cloud Computing (CC) applications and platforms. This will be used to provide a framework for organization to support strategy in mitigating digital security risk through the deployment and usage of digital innovations platforms and applications in the Banking Sector.

Your kind and objective responses will significantly contribute to the topic. Any information provided is strictly for academic purposes and would be treated with utmost confidentiality. This research has been reviewed for ethical appropriateness by the Ethics and Research Committee of the school. After completion, you can access the research results upon request.

Please, do not hesitate to contact me by email if you have queries, or seek clarifications.

Thank you

This part of the questionnaire is based on a **10-point linguist scale** used for evaluating ***Occurrence (O), Severity (S) and Detection (D)*** of vulnerabilities in a system as recommended by Goodman (1996), and used for FMEA applications (Lin et al., 2014; Lui et al., 2012).

Please indicate the level of risk failures mode and weights for each vulnerability and their likelihood of occurrence, severity and detection on each of the risk dimensions identified.

Severity (S) Rating Scales: the extent of failure mode affecting the entire system

Where 10- Extremely Vulnerable and 1 No Vulnerability

Security Dimension	Failure Mode	Rating									
	**Severity**	**1**	**2**	**3**	**4**	**5**	**6**	**7**	**8**	**9**	**10**
**Access to Information and digital platform**	User ID authentication failures										
	User register audit failures										
	External management access control										
	Management of removable of internal and external media risk										
	Control to third party privileges risk										
	Access to external digital platforms control										
**Network Security**	Failure of Firewall protection										
	Poor system connections										
	File transfer protocol to authenticate the communication between devices and networks										
	Lack of intrusion detection and prevention system										
	Lack of preventing network attacks										
**Digital Platform interactivity**	Digital Platforms compatibility failures										
	Reliability of digital platforms										
	Failure of software functionality on platforms										
	Failure of digital configuration with digital system										
	Lack of digital platform interactions										

**Infrastructure**	Failure to control use of infrastructure										
	Lack of infrastructure update and patching										
	Software origination and defence failures										
	Lack of information back-up										
	Lack of back-up electric generator										
	Lack of network node certification										
**Security Management**	Lack of information security audit										
	Lack of policy paper on digital security safety										
	Lack of maintenance of hardware and software										
	Lack of security policies reviews										
	Lack of responsibility to information safety										
**Identity Management**	Lack of securing users' true identity										
	Lack of identifying third party identity										
	Notification to system administrator on user's identity										
	Lack of detecting outsourced party activity and identity										
**Communication Security**	Lack of encryption control management										
	Lack of limited content access to internet										
	Lack of security safety education and training										
	Lack of safety of electronic mail										
	Lack of safety of electronic office systems										
**Data and Information Management**	Lack of data scalability										
	Failure to secure data transferability										
	Failure to provide data privacy										
	Lack of preventing data theft										
	Failure to prevent unauthorised use of data and information										
**Digital security systems development**	Lack of standardization and documentation of the software development process										
	Conflict in Software and vulnerability testing										
	Lack of a change monitoring log										
	Failure to update and maintain security system										
	Failure to test security risk management procedures										

Occurrence (O) Rating Scale-frequency of occurrence of the failure modes within a specified time period.

10- Certain probability of occurrence and 1- Remote probability of occurrence

**Security Dimension**	**Failure Mode**	**Rating**									
	**Occurrence**	**1**	**2**	**3**	**4**	**5**	**6**	**7**	**8**	**9**	**10**
**Access to Information and digital platform**	User ID authentication failures										
	User register audit failures										
	External management access control										
	Management of removable of internal and external media risk										
	Control to third party privileges risk										
	Access to external digital platforms control										
**Network Security**	Failure of Firewall protection										
	Poor system connections										
	File transfer protocol to authenticate the communication between devices and networks										
	Lack of intrusion detection and prevention system										
	Lack of preventing network attacks										
**Digital Platform interactivity**	Digital Platforms compatibility failures										
	Reliability of digital platforms										
	Failure of software functionality on platforms										
	Failure of digital configuration with digital system										
	Lack of digital platform interactions										

**Infrastructure**	Failure to control use of infrastructure										
	Lack of infrastructure update and patching										
	Software origination and defence failures										
	Lack of information back-up										
	Lack of back-up electric generator										
	Lack of network node certification										
**Security Management**	Lack of information security audit										
	Lack of policy paper on digital security safety										
	Lack of maintenance of hardware and software										
	Lack of security policies reviews										
	Lack of responsibility to information safety										
**Identity Management**	Lack of securing users' true identity										
	Lack of identifying third party identity										
	Notification to system administrator on user's identity										
	Lack of detecting outsourced party activity and identity										
**Communication Security**	Lack of encryption control management										
	Lack of limited content access to internet										
	Lack of security safety education and training										
	Lack of safety of electronic mail										
	Lack of safety of electronic office systems										
**Data and Information Management**	Lack of data scalability										
	Failure to secure data transferability										
	Failure to provide data privacy										
	Lack of preventing data theft										
	Failure to prevent unauthorised use of data and information										
**Digital security systems development**	Lack of standardization and documentation of the software development process										
	Conflict in Software and vulnerability testing										
	Lack of a change monitoring log										
	Failure to update and maintain security system										
	Failure to test security risk management procedures										

Detection (D) Rating Scale- the probability of detecting the failure
10- No chance of detection and 1- Almost chance of detection

**Security Dimension**	**Failure Mode**	**Rating**									
	**Detection**	**1**	**2**	**3**	**4**	**5**	**6**	**7**	**8**	**9**	**10**
**Access to Information and digital platform**	User ID authentication failures										
	User register audit failures										
	External management access control										
	Management of removable of internal and external media risk										
	Control to third party privileges risk										
	Access to external digital platforms control										
**Network Security**	Failure of Firewall protection										
	Poor system connections										
	File transfer protocol to authenticate the communication between devices and networks										
	Lack of intrusion detection and prevention system										
	Lack of preventing network attacks										
**Digital Platform interactivity**	Digital Platforms compatibility failures										
	Reliability of digital platforms										
	Failure of software functionality on platforms										
	Failure of digital configuration with digital system										
	Lack of digital platform interactions										
**Infrastructure**	Failure to control use of infrastructure										

	Lack of infrastructure update and patching										
	Software origination and defence failures										
	Lack of information back-up										
	Lack of back-up electric generator										
	Lack of network node certification										
**Security Management**	Lack of information security audit										
	Lack of policy paper on digital security safety										
	Lack of maintenance of hardware and software										
	Lack of security policies reviews										
	Lack of responsibility to information safety										
**Identity Management**	Lack of securing users' true identity										
	Lack of identifying third party identity										
	Notification to system administrator on user's identity										
	Lack of detecting outsourced party activity and identity										
**Communication Security**	Lack of encryption control management										
	Lack of limited content access to internet										
	Lack of security safety education and training										
	Lack of safety of electronic mail										
	Lack of safety of electronic office systems										
**Data and Information Management**	Lack of data scalability										
	Failure to secure data transferability										
	Failure to provide data privacy										
	Lack of preventing data theft										
	Failure to prevent unauthorised use of data and information										
**Digital security systems development**	Lack of standardization and documentation of the software development process										
	Conflict in Software and vulnerability testing										
	Lack of a change monitoring log										
	Failure to update and maintain security system										
	Failure to test security risk management procedures										
